# Stromal cells control soluble material and cellular transport in lymph nodes

**DOI:** 10.3389/fimmu.2012.00304

**Published:** 2012-09-27

**Authors:** Marc Bajénoff

**Affiliations:** ^1^Centre d’Immunologie de Marseille-Luminy, Parc Scientifique et Technologique de Marseille Luminy, Aix-Marseille University, UM2Marseille, France; ^2^Institut National de la Santé et de la Recherche Médicale, U1104Marseille, France; ^3^Centre National de la Recherche Scientifique, UMR 7280Marseille, France

**Keywords:** stroma, lymph node, soluble material transport, cellular migration

## Abstract

Lymphocytes continuously patrol the secondary lymphoid organs (SLOs) of mammals in search for their cognate antigens. SLOs are composed of leucocytes (~95%) and lymphoid stromal cells (~5%) that form the structural framework of these organs. These sessile cells have been considered for decades as inert elements of the immune system. This simplistic view has dramatically changed in recent years, when it was discovered that these architectural cells are endowed with immuno-regulatory functions. Lymph nodes (LNs) are located at the interface between the blood and lymphatic systems, thus allowing tissue-derived antigen/antigen presenting cells (APCs) to gather with blood-derived lymphocytes. As a typical LN contains ~10 million of tightly packed cells, this accumulation of immune cells and information is probably not sufficient to foster the rare cellular interactions mandatory to the initiation of adaptative immune responses. Herein, I review some of the physicochemical elements of stromal cells that are used to transport and guide immune cells and soluble molecules within LNs.

## TRANSPORTATION OF IMMUNE INFORMATIONS

Lymphatics continuously transport soluble and particulate Ags from peripheral tissues to draining lymph nodes (LNs; Young, 1999; Willard-Mack, 2006). This lymphatic content reflects the immunological status of peripheral tissues and is constantly deciphered by antigen presenting cells (APCs) and lymphocytes within LNs. Afferent lymphatics discharge their content in the LN subcapsular sinus (SCS), a hollow tubular structure that surrounds the LN, thus preventing free diffusion of the lymphatic content to the underlying parenchyma (Forkert et al., 1977; van Ewijk et al., 1988; Willard-Mack, 2006). The vast majority of APCs and lymphocytes reside in the enclosed LN parenchyma from which free soluble particles are excluded. This structural confinement raises a critical question: how is soluble and particulate material transported from the SCS throughout the parenchyma?

### SCS MACROPHAGES

**FIGURE 1:**
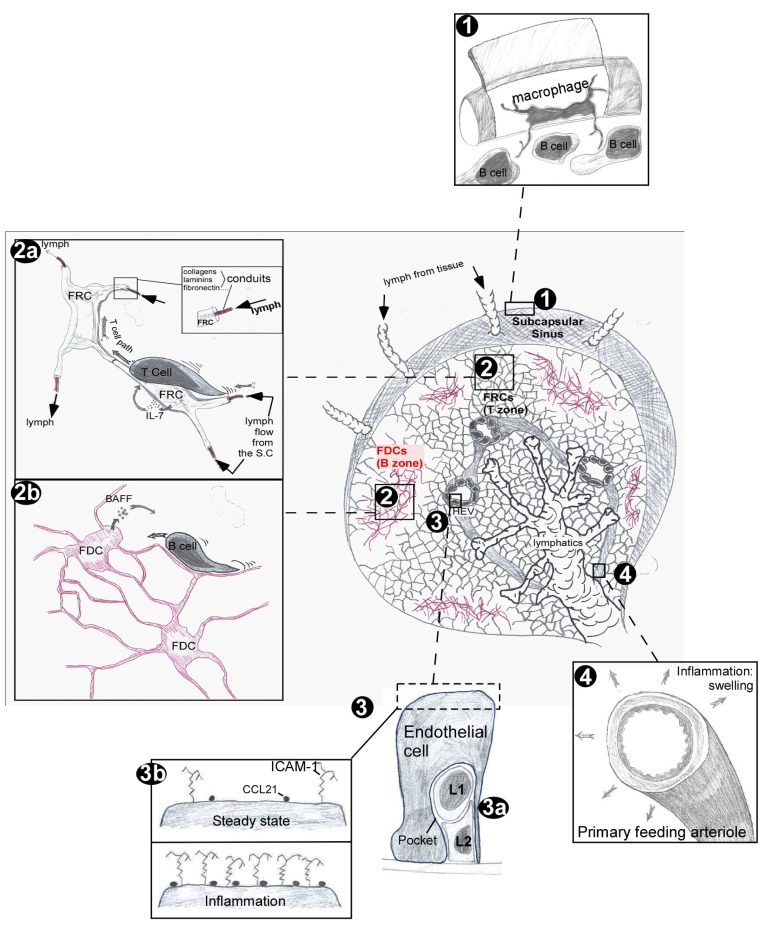
**Stromal cell guided transport of soluble material and immune cells within lymph nodes**.

The floor of the SCS is composed of a layer of sinus endothelial cells and a layer of specialized fibroblasts (Forkert et al., 1977; Farr et al., 1980). The integrity of the floor of the SCS is a subject of conflicting reports. Ultrastructural studies demonstrate pores in the floor of the SCS by electron microscopy (Forkert et al., 1977; van Ewijk et al., 1988) while others argue against such evidences (Farr et al., 1980). Despite the putative existence of pores in the floor of the SCS, there is evidence that penetration of particulate material from lymph into LN cortex is limited (Gretz et al., 1997). SCS is populated by a subpopulation of SCS macrophages that extend cytoplasmic protrusions to the underlying B cell follicle. Intravital imaging of the SCS in live animals demonstrated macrophage capture of particulate antigen and transfer to Ag-specific B cells via these protrusions (Carrasco and Batista, 2007; Junt et al., 2007; Phan et al., 2007; **Figure 1**, item 1). Further experiments demonstrated that complement receptors 1 and 2 expression on B cells is important for the capture and delivery of immune complexes from SCS macrophages to germinal centers (GCs) and follicular dendritic cells (FDCs; Phan et al., 2007, 2009). Therefore, SCS macrophages act as Ag-bridging channels between the impermeant SCS and B cell follicles.

### THE CONDUIT SYSTEM

The SCS is a shielded unit that prevents the free diffusion of particulate Ags and soluble material >70 kDa to the parenchyma (Gretz et al., 2000). Tissue-derived cells such as dendritic cells (DCs) can actively cross the layer of SCS-lining cells (Braun et al., 2011) whereas chemokines, interleukins (ILs), and small Ags can diffuse to the parenchyma via a dedicated network of pipes named conduits (Sainte-Marie and Peng, 1986). Conduits originate between the SCS-lining cells (Gretz et al., 1997) and are found throughout the paracortex, mainly within the T cell zone. These reticular fibers are composed of several layers of extracellular matrix molecules precisely assembled around a core of collagen fibers (refer to Sixt et al., 2005; Roozendaal et al., 2008 for an extensive description of the conduits composition). Conduits are produced and ensheathed by Fibroblastic reticular cells (FRCs; Gretz et al., 1997; Katakai et al., 2004b; **Figure 1**, item 2a) and as a result, most of the conduit system is shielded from lymphoid and myeloid cells within the T cell zone. Despite its physical enclosure, many immune cell types capture soluble material from the lymphatic content conveyed by the conduit network.

#### High endothelial venules

High endothelial venules (HEVs) are the gateways intravascular lymphocytes use to immigrate into the LN parenchyma from the blood circulation (Cho and De Bruyn, 1986; Girard and Springer, 1995). Lymphocyte recognition of HEVs involves a multistep adhesion cascade in which primary adhesive interactions (rolling) are followed by firm arrest (sticking) in response to chemokines (Sasaki et al., 1996). The endothelial cells of HEVs secrete and present chemokines such as CCL21 on their luminal surface (Gunn et al., 1998). As most of the tubules of the conduit system end in HEVs, the luminal surface of these structures is also rapidly decorated by lymph borne chemokines transported via the conduit system (Gretz et al., 2000; Baekkevold et al., 2001; Palframan et al., 2001). Therefore, the conduit system acts as a remote messenger system able to modulate lymphocytes trafficking across LN HEVs.

#### Resident dendritic cells

Lymph nodes contain an important population of resident DCs that settle on the FRC network (Sixt et al., 2005; Bajenoff et al., 2006) and are capable of taking up and processing soluble antigens transported within the conduits (Sixt et al., 2005).

In steady state conditions, these Ag-loaded DCs present peptide/MHC complexes to T cells in absence of co-stimulatory molecules and should hence promote peripheral tolerance (Probst et al., 2003, 2005). Upon infection, inflammatory stimuli, soluble Ag, and tissue-derived DCs loaded with pathogen peptide/MHC complexes are drained to the proximal LN (Itano et al., 2003). In these conditions, resident DCs probably present tissue-derived Ags in a pro-inflammatory environment susceptible to initiate adaptive immune responses (Itano et al., 2003).

The important meshwork of resident DCs may represent a very efficient way to “deploy” the antigenic repertoire conveyed by the conduit system. Such widespread antigenic representation may ensure an optimal scanning of lymphocytes during their journey in the LNs, both in steady state and inflammatory conditions.

#### B cells and follicle dendritic cells

Although the conduit system is synthesized by T cell zone FRCs, sparse conduits are present in B cell follicles. Like their T cell counterparts, follicular conduits convey soluble (but not particulate) material from the SCS throughout B cell follicles. Using two-photon (2P) technology, Roozendaal et al. (2009) observed that conduits deliver small antigens and chemokines such as CXCL13 to B cells that directly contact the conduits. Another study demonstrated that the conduit system is also used to deliver soluble Ag to FDCs (Bajenoff and Germain, 2009).

#### Efficiency of the transport

Subcutaneous injection of fluorescent tracers demonstrated that the transportation of soluble material from the peripheral tissue to the parenchyma of the draining LN occurs within minutes (Itano et al., 2003; Roozendaal et al., 2009). The efficacy of the conduit system is quite surprising given (i) the numerous resident DC processes supposedly stuck in narrow conduits, (ii) the complex 3D branching pattern of the conduit system, and (iii) the absence of identified lymph propelling system. Further experiments will be required to understand the fine details that control lymph propulsion within these micropipes.

## TRANSPORTATION OF CELLS

### CONTROL OF LYMPHOCYTE FLUX

#### Steady state

Millions of lymphocytes enter and exit LNs each day, accessing the parenchyma via HEVs and egressing via efferent lymphatics. Despite this high rate of cellular flux, the number of lymphocytes present in a resting LN is extraordinary stable over time. The control of lymphocyte trafficking is mediated by the endothelial cells of HEVs that harbor typical cobblestone shapes with numerous embedded lymphocytes (Girard and Springer, 1995). Recent evidences revealed that these T and B cells are frequently packed together underneath the endothelial cell inside “pockets” composed of 4–5 lymphocytes (**Figure 1**, item 3a). These pockets function as waiting areas that hold and grant lymphocytes access to LN parenchyma in proportion to the rate of lymphocyte egress from the LN, enabling the LN to maintain a constant cellularity while supporting the extensive cellular trafficking necessary for repertoire scanning (Mionnet et al., 2011).

#### Inflammation

Lymph nodes are highly vascularized structures that, upon inflammation, can remodel and expand their primary feed arterioles by 50%, leading to a four- to fivefold increase in the rate of naive lymphocyte flow rate through the draining LNs (**Figure 1**, item 4; Soderberg et al., 2005). At the same time, the pro-inflammatory mediators released from the inflammatory site are transported via the conduits to the HEVs of the draining LNs (Baekkevold et al., 2001). IL-6 increases intercellular adhesion molecule-1 (ICAM-1) expression on HEVs, thereby promoting lymphocyte adherence to HEVs of the draining LN. This phenomenon may also apply to IL-8 and tumor necrosis factor (TNF-α) that have been shown to rapidly increase T cell entry into the draining LN (Larsen et al., 1989; McLachlan et al., 2003). In addition, memory and effector T cells that lack CD62L expression rapidly gain entry into inflamed LNs through expression of CXCR3 and its interactions with CXCL9 deposited on the luminal surface of inflamed HEVs (Wurtz et al., 2004). Finally, temperatures ranging from 38–40 ºC act directly on lymphocytes to enhance CD62L-dependent homing across HEVs while also increasing the expression of CCL21 and ICAM-1 on the surface of HEVs (**Figure 1**, item 3b; Chen et al., 2006).

Altogether, these results present HEVs as gatekeepers in charge of modulating lymphocyte trafficking to LNs, both at steady state and during inflammation.

### CONTROL OF LYMPHOCYTES MOTILITY AND TERRITORIALITY

Within SLOs, T and B cells are highly mobile and segregate in distinct geographical areas populated by different stromal cells (Miller et al., 2002). FRCs reside in the T cell zone while FDCs populate B cell follicles (Gretz et al., 1997; Allen and Cyster, 2008; Mueller and Germain, 2009). Both stromal cell populations form dense, intermingled 3D networks in their respective areas (Schneider and Tschopp, 2003; Bajenoff et al., 2006; Munoz-Fernandez et al., 2006; Link et al., 2007; Allen and Cyster, 2008).

#### Fibroblastic reticular cells

Fibroblastic reticular cells are fibroblast-like cells that reside in the T cell area of LNs and spleen. FRCs produce and enwrap the conduit system, forming a rigid cellular network embedded amongst motile lymphocytes (Anderson and Anderson, 1976; Gretz et al., 1997; Sixt et al., 2005). Intravital two-photon (2P) imaging experiments have revealed that the FRC network supports and guides T and B cell motility in the T cell area (Bajenoff et al., 2006), dictating the apparent characteristic random migratory behavior of these cells. Lymphocytes follow the supporting fibers of the FRC as they migrate in the T cell zone that is itself defined by the extent of this network (**Figure 1**, item 2a).

The molecular cues that drive lymphocyte locomotion on FRCs have partially been deciphered. FRCs secrete the homeostatic chemokine CCL21 that stick to collagen IV and glycosaminoglycans (GAGs) present on the surface of FRCs (Ansel et al., 2000; Sixt et al., 2005; Link et al., 2007; Yang et al., 2007). CCR7, the receptor for CCL21, is expressed by many cell types, including DCs, T and B lymphocytes (Yanagihara et al., 1998; Forster et al., 1999). Both molecules are critical for the proper delimitation of the T/B cell boundary within SLOs as evidenced by the inability of T and B cell areas to properly segregate in the SLOs of CCL21 and CCR7 deficient mice (Nakano et al., 1998; Forster et al., 1999). Three recent dynamic imaging studies demonstrated that CCR7/CCR7-L signaling pathway is a key modulator of T cell locomotion (Asperti-Boursin et al., 2007; Okada and Cyster, 2007; Worbs et al., 2007). These studies concluded that the effect of CCR7 or CCR7-ligand deficiency could account for ~ 40% of the Gi-dependent motility of T cells in LNs.

Altogether, these results indicate that the random locomotion of T cells in LNs is physically and chemically guided by FRCs while CCR7/CCR7-ligands modulate the velocity of T cells. The exact set of molecular/chemical cues that regulate T cell migration remains to be determined.

#### Follicular dendritic cells and follicular stromal cells

Follicular dendritic cells present native antigens in the form of immune complexes on their surface and are critical for the maintenance of B cell follicle integrity (Cyster et al., 2000; Allen and Cyster, 2008; Wang et al., 2011). Recent evidence indicate that FDCs arise from ubiquitous perivascular precursors (preFDC) expressing platelet-derived growth factor receptor β (PDGFRβ; Krautler et al., 2012). During immune responses, FDCs organize the development of GCs in which mature B lymphocytes rapidly proliferate, differentiate, mutate their antibodies through somatic hypermutation, and class switch their antibodies (MacLennan, 1994; Allen et al., 2007a; Allen and Cyster, 2008; Wang et al., 2011). Intravital 2P experiments have revealed that B cells migrate on the thin and intermingled processes of radio-resistant stromal cells populating B cell follicles, suggesting that FDCs are the counterparts of FRCs in the B cell follicles (Bajenoff et al., 2006; **Figure 1**, item 2b).

Follicular dendritic cells are defined by their capacity to trap and retain immune-complexes and their expression of various markers such as CD21/35 (complement receptors 1 and 2) and C4 complement fraction (Cyster et al., 2000). FDCs also express BP3, a glycosylphosphatidyl-anchored membrane protein (McNagny et al., 1991) of unknown function. Surprisingly, BP3 staining in B cell follicle highlights a non-FDC network, suggesting the existence of a second follicular stromal cell network (McNagny et al., 1991; Allen and Cyster, 2008). Therefore, it is likely that both FDCs and these radio-resistant follicular stromal cells support B cell migration in primary B cell follicles. Further experiments will be required to address this issue.

Follicular dendritic cells and follicular stromal cells are an important source of CXCL13 in follicles and this chemokine is known to promote B cell migration *in vitro* and organize B cell follicle formation *in vivo* (Legler et al., 1998; Ansel et al., 2000; Saez de Guinoa et al., 2011). Two-photon microscopy analysis of GC B cell motility showed that it was reduced in the absence of CXCL13 suggesting that this chemokine may also promote B cell motility in primary follicles (Allen et al., 2007b).

### OPEN QUESTIONS

#### Control of lymphocyte trafficking on FRC and FDC networks

T and B cells actively migrate on stromal cell networks, adapting their paths to the 3D processes of these supporting cells. Such stochastic behavior may ensure that a given lymphocyte will eventually visit its entire territory before leaving the LN. However, LNs are densely packed organs in which extracellular space is limited, if not absent. Therefore, wandering lymphocytes should constantly “bump” to each other during their random migration. Interestingly, we consistently observed that T cells never turned back in the middle of a FRC fiber but always changed direction at FRCs intersections (personal observation). It would then be interesting to determine how lymphocytes move as a population and whether they constantly bump and squeeze on each other or line up along stromal cells during their migration.

#### Stromal cell behavior in inflamed LNs

Lymph nodes draining an inflamed tissue rapidly enlarge in response to the massive influx of naive cells and the proliferation of the activated ones, probably inducing a tremendous and rapid remodeling of the various stromal cell subsets that should not only continue to fulfill their steady state duties but also create new microenvironments necessary for the development of the immune response (e.g., GCs, medullary cords, etc.; Katakai et al., 2004a,b; Allen et al., 2007a; Allen and Cyster, 2008). So far, we do not understand how LN stromal cells manage these rapid structural changes and cellular demands.

***Are inflamed stromal cells able to stretch?*** Fibroblastic reticular cells and FDCs form 3D substrata for lymphocytes. Upon inflammation, these networks should accommodate the massive influx of lymphocytes and continue to generate cellular roads for them. FRCs and FDCs express contractile molecules normally restricted to smooth muscles (desmin, smooth muscle actin, etc.) and myofibroblasts, a subset of activated fibroblasts capable of speeding wound repair by contracting the edges of the wound (Sixt et al., 2005; McAnulty, 2007). FRCs are also endowed with contractile properties as evidenced by their capacity to induce wrinkles on deformable collagen-coated silicone substrate (Link et al., 2007). As FRCs are attached to collagen-rich conduits, these properties may allow them to stretch in order to increase their surface and accommodate the massive influx of T cells consecutive to inflammation. Their contractile properties may also be used to shrink the conduits upon the completion of the immune response in order to restore the original size of the LN. The calculation of FRC and FDC densities as well as the precise measurement of their dimensions in resting and inflamed LNs may test these hypotheses.

***Origin of additional stromal cells in inflamed LNs.*** Inflamed LNs can triple their size in few days and undergo a tremendous enlargement in chronically infected mice (Webster et al., 2006; Ruddle and Akirav, 2009). It is thus likely that FRC and FDC networks incorporate new stromal cells in order to sustain this remodeling. The origin of lymphoid stromal cells remains elusive, though there are growing evidences that they are of mesenchymal origin (Munoz-Fernandez et al., 2006; Mabbott et al., 2011). In addition, the SLOs of irradiated hosts reconstituted with syngeneic bone marrow cells possess lymphoid stromal cells of host origin (Humphrey et al., 1984; Bajenoff et al., 2006). These observations led to the conclusion that adult lymphoid stromal cells do not originate from bone marrow mesenchymal cells at steady state. However, these conclusions should be interpreted with caution. Bone marrow hematopoietic stem cells only engraft when adoptively transferred in an irradiated host, demonstrating that the destruction of pre-existing hematopoietic cells/progenitors is a prerequisite for the engraftment of hematopoietic progenitors. Stromal cells and their progenitors are radio-resistant. Therefore, if grafted bone marrow cells contain mesenchymal stromal cell progenitors, these cells will fail to engraft, even when adoptively transferred in an irradiated host. In absence of prior stromal cell destruction, any adoptive transfer of stromal cell progenitor is probably destined to fail. The determination of the origin of stromal cells in resting and inflamed LNs will probably require the creation of new animal models that are currently critically lacking in the field of stromal cell biology.

## Conflict of Interest Statement

The author declares that the research was conducted in the absence of any commercial or financial relationships that could be construed as a potential conflict of interest.
